# "I don't want my marriage to end": a qualitative investigation of the sociocultural factors influencing contraceptive use among married Rohingya women residing in refugee camps in Bangladesh

**DOI:** 10.1186/s12978-024-01763-8

**Published:** 2024-03-07

**Authors:** Mansura Islam, Shah Ehsan Habib

**Affiliations:** 1 School of General Education, BRAC University, Dhaka 1212, Bangladesh; 2 Department of Sociology, University of Dhaka, Dhaka 1000, Bangladesh

**Keywords:** Modern contraception, Sociocultural factors, Rohingya women: Refugee camps

## Abstract

**Background:**

The timely provision of comprehensive contraceptive services to Rohingya women is impeded due to a lack of clarity and understanding of their traditional beliefs and cultural frameworks. Recognizing this challenge, our paper aims to explore the socio-cultural factors influencing the utilization of contraceptives among married Rohingya women living in the refugee camps of Cox's Bazar, Bangladesh.

**Method:**

A qualitative study was conducted in two unregistered Rohingya camps (Camp 7&14) located in Ukhiya Upazila, Cox's Bazar from January 10th to 20th, 2022. A total of 14 In-Depth Interviews (IDIs) were conducted among married Rohingya women of reproductive age (15–49 years), along with 16 Key Informant Interviews (KIIs) involving stakeholders engaged in reproductive healthcare provision. Participants were selected using purposive sampling. All interviews were conducted in the local language, recorded, transcribed verbatim, and subsequently translated into English. The data were analyzed using NVivo (Version 11), and the analysis process followed Neuman’s three-phase coding system.

**Results:**

Five broad themes were identified: Sociocultural expectations and values attached to births, power imbalances within marital relationships, the role of religious beliefs, fear of side effects, and misperceptions about contraception. Having a larger number of children is viewed positively as it is believed that children play a crucial role in preserving the lineage and contributing to the growth of the Islamic population. Despite expressing an inclination towards contraception, the disapproval of husbands becomes a significant barrier for women. Defying their husbands' wishes can result in instances of Intimate Partner Violence (IPV) and even marriage dissolution within the camps. Moreover, the fear of side effects, such as a particular method would cause infertility, discourages women from using contraception. Many of these fears stem from myths, misconceptions, and mistrust in the existing medical system.

**Conclusion:**

Addressing the socio-cultural barriers that prevent women from using modern contraception will have important public health implications. These findings can support in crafting culturally sensitive programs and educational interventions. These initiatives can assist Rohingya refugee women in planning their pregnancies and reducing high-risk pregnancies, ultimately leading to a decrease in maternal mortality rates within the community.

## Plain language summary

The timely provision of comprehensive contraceptive services to Rohingya women is impeded due to a lack of clarity and understanding of their traditional beliefs and cultural frameworks. Taking this into account, our objective is to investigate the socio-cultural factors that influence the utilization of contraceptives among married Rohingya women living in the refugee camps of Cox's Bazar, Bangladesh. A total of 14 IDIs and 16 KIIs were conducted in two unregistered Rohingya camps. We found that contraceptive use among Rohingya refugees was constrained by various socio-cultural and religious beliefs. The desire for a larger number of children to ensure the continuity of the lineage and to be able to contribute to the growth of the Islamic population serves as a major barrier. Lack of decision-making power regarding reproductive life not only stops participation but also makes women vulnerable to IPV and marriage dissolution within the camps. Moreover, the fear of side effects, such as a particular method would cause infertility, discourages women from using contraception. Many of these fears stem from myths, misconceptions, and mistrust in the existing medical system. Young women often acquire information about both true side effects and myths from their social networks. Therefore, developing culturally sensitive programs and educational interventions targeting the women, their peers, and other related communities is indispensable to assist Rohingya refugee women in overcoming the identified barriers to contraception.

## Introduction

Comprehensive family planning support is an essential requirement for refugees [1]. In situations of displacement, refugee women face increased risks of unintended pregnancies, insufficient intervals between childbirths, adverse outcomes due to unsafe abortions, and elevated rates of maternal mortality [2, 3]. Moreover, displaced women are highly susceptible to sexual violence, exploitation, and abuse, which can lead to various health complications and the transmission of incurable infections such as HIV [2, 4, 5]. To prevent such adverse outcomes, contraception use is strongly recommended and acknowledged as an essential component of primary sexual and reproductive health interventions in humanitarian crises [6]. Recognizing the advantages and acknowledging the life-threatening nature of these hazards, the International Conference on Population and Development (ICPD), held in Cairo in 1994, integrated the provision of comprehensive family planning services into the broader framework of reproductive health [7, 8].

Globally, there has been a concerning rise in the number of refugee population. According to estimates from the United Nations High Commissioner for Refugees (UNHCR), the number of refugees who had crossed international borders reached approximately 32.5 million as of mid-2022 [9]. This figure includes around 1 million Rohingya refugees who have been residing in Cox's Bazar District of Bangladesh since 1970 [10]. The most significant and rapid influx occurred in 2017, when over 700,000 Rohingya fled to Cox's Bazar due to persecution, discrimination, and targeted violence [11]. The majority of Rohingya refugees consist of women and girls, making up more than 50% of the population, with women of reproductive age accounting for 24.3% of the total [12]. Evidence indicates that deaths among Women of Reproductive Age (WRA) and neonatal deaths make up 28% of the overall recorded deaths in the camps [13]. Furthermore, around 31.3% of WRA deaths were attributed to pregnancy, childbirth, and postpartum complications, commonly referred to as maternal deaths [13]. This situation has been linked to inadequate health-seeking behavior, including low utilization and a low prevalence of contraceptive methods among the population [1, 6, 13].

To effectively address the reproductive and maternal health concerns, a total of 35 stakeholders, comprising government bodies and other humanitarian actors are collaborating to facilitate the delivery of Sexual and Reproductive Health and Rights (SRHR) services, including Family Planning (FP). These services are being delivered through more than 173 healthcare facilities located in Rohingya refugee camps and host communities [13]. The effective coordination among these stakeholders, coupled with various awareness-raising initiatives, has resulted in an increase in contraceptive prevalence among Rohingya women. The prevalence has risen by 2.1 percentage points, from 33.7% in 2018 to 35.8% in 2019 [14]. The prevalence of contraceptive use is notably higher among women engaged in income-generating activities, who sought medical assistance at healthcare facilities within the camp and primarily relied on physicians or nurses for their knowledge regarding family planning [15, 16]. However, actors involved in the initiatives reported encountering significant challenges arising from religious and cultural values, as well as deep-rooted misconceptions regarding contraceptive use among the majority of the Rohingya people [6, 13]. The prevailing patriarchal social structure in this community has disempowered women, restricting their involvement in decision-making processes concerning their reproductive lives. Furthermore, despite the efforts of several organizations to provide family planning (FP) services, Rohingya women and girls still face inadequate and unequal access to these crucial services [15].

The sociocultural context in which Rohingya people are raised limits the provision for the reproductive health services, including family planning in the refugee camps. While there is scant literature investigating the socio-cultural practices of this community, it notably lacks exploration into how these practices affect contraceptive utilization [14, 16–19]. With this in consideration, our goal is to investigate the socio-cultural factors that influence the utilization of contraceptives among married Rohingya women living in the refugee camps of Cox's Bazar, Bangladesh. The findings from this research will aid in the development of a comprehensive guideline for culturally sensitive interventions. This study will play a pivotal role in improving overall sexual and reproductive health standards and empowering girls and women to actively engage in decisions concerning their reproductive lives.

## Methods

### Study design

A qualitative study was conducted, involving In-depth Interviews (IDIs) and Key Informant Interviews (KIIs). IDIs were conducted among married Rohingya refugee women of reproductive age (15–49 years), and KIIs were conducted with stakeholders related to different sections of reproductive healthcare facilities. The selection of this qualitative method aimed to provide an in-depth explanation of prevailing perceptions and socio-cultural factors influencing contraceptive use.

### Study setting

The study was conducted in two unregistered Rohingya camps: Kutupalong (camp 7) and Hakim Para (camp 14), located in Ukhiya Upazila, Cox's Bazar. These two camps were established due to the influx of approximately 700,000 Rohingya in 2017, seeking refuge in various camps within Cox's Bazar [11]. Camp 7 covers an area of 714,846 square meters, with a population density of 19.5 square meters per person. The total population of the camp is approximately 36,646, with females accounting for 52% of the population. Among them, there are 8,973 reproductive-age females [20]. On the other hand, Camp 14 spans an area of 857,724 square meters, with a population density of 27.1 square meters per person. The total population of the camp is approximately 31,653, with females comprising 51% of the population. The number of reproductive-age females in Camp 14 is 7,416 [21].

### Study participants and sampling

A total of 16 KIIs and 14 IDIs were conducted (Table 1). To be eligible for the IDIs, women had to meet the following criteria: (1) married, (2) fall within the reproductive age range of 15–49 years, (3) reside in unregistered refugee camps, and (4) possess knowledge about contraceptive methods. In order to capture diverse perspectives, stakeholders such as Community Health Workers (CHWs), field supervisors, midwives, doctors, and information management officers were selected for the KIIs. The selection of participants for both KIIs and IDIs followed a criterion-based purposive sampling procedure.

**Table 1:** Distribution of the sample by camp, Cox's Bazar

Setting	KII	IDI	Total
Camp 7	Community Health Worker—2 Midwife—1 Medical Officer—2 Supervisor of CHWs—2 Information Management Officer—1	Married Rohingya women of reproductive age—7	15
Camp 14	Community Health Worker—2 Midwife—1 Medical Officer—2 Supervisor of CHWs—2 Information Management Officer—1	Married Rohingya women of reproductive age—7	15
Total	16	14	

Note: KII= Key Informant Interviews, IDI= In-depth Interviews

### Data collection

The data collection took place from January 10th to 20th, 2022. Five female data collectors with prior field data collection experience were recruited and trained by the primary investigator. These data collectors were selected based on their profession as primary school teachers and their proficiency in the language of Rohingya, which is similar to the Chittagonian language. Their profession also provided the advantage of being well-known within the community, fostering a friendly atmosphere during data collection. This familiarity facilitated respondents' comfort and flexibility in discussing sensitive topics such as contraceptive use, marriage, sexual life, pregnancy, and related matters. However, steps were taken to minimize biases and maintain objectivity. Training sessions emphasized the importance of neutrality, confidentiality, and ensuring a non-judgmental approach during interviews. Additionally, respondents were assured of the confidentiality of their responses.

An open-ended semi-structured interview guide was used to explore attitudes and barriers related to the utilization of contraceptive facilities provided by healthcare centers. To ensure consistency, the interview guide was pretested with four participants who were not part of the study sample. After conducting pretesting, changes were made, such as simplifying language for participants and removing few sensitive questions for instance- "Have you ever experienced sexual violence and how has it affected your contraceptive choices?".

The IDIs were conducted individually and face-to-face at the respondent's residence, while the KIIs took place in a private room within the healthcare centers. The duration of the interview sessions ranged from 20 to 50 min, with an average duration of 30 min. The IDIs were conducted in the Rohingya language and later translated into English. On the other hand, the KIIs were conducted in Bangla and also translated into English. Audio recordings of each interview were made with the participant's permission.

The interviews began by gathering the participants' demographic details, after which they were asked questions regarding the following topics: reasons for using or not using modern contraceptives, and factors that hinder Rohingya women from utilizing modern contraception. Data collection concluded when thematic analysis revealed saturation, indicating redundancy in information and the absence of novel insights on the discussed topics.

### Data analysis

The data obtained from the IDIs and KIIs were transcribed verbatim and then translated into English by the data collector. To ensure accuracy, the transcripts underwent a validation process involving two separate groups of transcribers reviewing them. The transcripts were uploaded in NVivo (Version 11) for analysis. The analysis was done independently by the researchers and guided by Neuman’s three-phase coding system, previously applied in comparable contexts [22–24]. In the first phase, the investigators read the transcribed data multiple times to familiarize themselves with the factors considered and gain an in-depth understanding of the text. During this process, codes and sub-codes were generated, identified, and defined within broader categories. In the next phase, the investigators examined the codes and organized the data to identify significant patterns of meaning or potential themes. This involved a detailed analysis focusing on informative names for each theme. The identified themes were then verified, cross-checked, and thoroughly discussed among the investigators to ensure that the study's objective was achieved. To validate the key findings of the study, information obtained from the Rohingya women and key informants was triangulated, meaning that multiple data sources were compared and analyzed together. This process helped enhance the credibility and reliability of the study's findings.

## Results

### Background characteristics

Table 2 presents the detailed characteristics of the IDI respondents. Their ages ranged from 19 to 40 years, with an average age of 27 ± 5.5 years. All respondents were married, possessed knowledge about modern contraception, and identified as Muslim. Out of the respondents, only 2 were literate, having completed primary education. The average age of marriage was 14 ± 0.88 years, and the average household size was 6 ± 1.29 members.

**Table 2:** Characteristics of the IDI participants

Characteristics	IDI		
	Number	Camp 7	Camp 14
*Age (n* = *14)*			
19–23	3	2	1
24–28	6	3	3
29–33	3	1	2
34–38	1	1	0
39 +	1	0	1
*Education (n* = *14)*			
Illiterate	12	6	6
Literate	2	1	1
*Age of marriage (n* = *14)*			
13	4	2	2
14	7	4	3
15	2	1	1
16	1	0	1
*Household members (n* = *14)*			
3–5	6	4	2
6–8	8	3	5

Note: IDI= In-depth Interviews

### Key themes

Five key themes emerged during the analysis, encompassing the perspectives of both Rohingya women and stakeholders. These themes include: sociocultural expectations and values attached to births, power imbalances between spouses, role of religious beliefs, fear of side effects, and misperceptions about contraception. Each theme sheds light on important aspects of the issue at hand.

### Sociocultural expectations and values attached to births

Among Rohingya women, a prevailing belief exists that their primary purpose of life is to give birth and fulfill the responsibilities of caring for their husbands and children. The respondents have observed how traditions have consistently led their mothers and grandmothers to prioritize the desires of their families over their own needs. This belief and behavior have been transmitted across generations.

As women, we are inherently tasked with nurturing our families and children. In my own experience, I grew up with 6 siblings, and my mother was responsible for our care. I, in turn, raised 4 daughters who are now married and devotedly caring for their husbands and children.

(A 40-year old Rohingya woman, Camp 14)

Women are expected to conceive shortly after getting married, leading to the majority of them having their first child within the initial year of marriage. A notable proportion of the respondents expressed a preference for having children, particularly sons. The primary rationale for desiring additional children is rooted in the perception of children as a source of strength, which can be interpreted in multiple ways. Firstly, children are perceived as a wellspring of physical strength, as respondents highlighted the physical capabilities of males in terms of carrying heavy objects and enduring prolonged periods of physical labor. Secondly, children are seen as a source of economic strength and social security for the family. Lastly, children possess the capacity to uphold a lineage, ensuring its continuation.

I have four daughters. I conceived my 1^st^ daughter immediately after my marriage. I and my husband are trying to conceive again, with the hope of having a son this time. My husband is old and the only person who looks after us. Who will look after the family and my daughters after his death? Daughters get married and move to their husbands' homes, while sons typically remain with their parents.

(A Rohingya mother of four daughters, Camp 14)

The findings from KIIs further validated the insights obtained from the IDIs. Children are perceived as valuable assets within the Rohingya community. KII respondents illustrated that having more children resulted in increased food assistance from NGOs. This surplus food is often sold in local markets at lower prices to generate income. Consequently, Rohingya individuals do not fret over securing food for an additional family member; rather, they hold the belief that an additional member will augment the food supply. This inclination significantly influences their decisions regarding contraceptive use.

One of the most practical reasons for having children is food cards. The food/ ration cards are distributed considering the number of family members. The more the member will be, the more the food card they will get. The difference between food cards and ration cards is that food card is given by WFP (World Food Program) and ration card is given by different NGOs. Now they get enough food from NGOs and they have no tension at all that how they will feed their children.

(Statement from a supervisor of CHWs in Camp 7)

An increased number of children equates to more available manpower within this community. Given the losses they have suffered in terms of community members, relatives, and neighbors, they hold the belief that a larger number of children will assist in the community's reconstruction. Moreover, in addition to community preservation, having children is perceived as a strategy for upholding the family lineage.

To them, having only one child equates to having no child at all. Some even go to the extent of marrying 3 to 4 times in pursuit of having offspring. They firmly believe that a larger family translates to enhanced empowerment. The greater the number of family members, the stronger the family unit becomes; conversely, fewer members signify weakness.

(Statement from a Field Supervisor, Camp 7)

### Power imbalances within marital relationships

Strong cultural norms and religious values dictate behavior, including reproductive choices, in the humanitarian context of Cox's Bazar. One of these norms grants male family members the authority to make all household decisions. These patriarchal values promote women's subordination to men, leading to power imbalances within marriages and limiting women's autonomy in deciding whether to use contraception.

As the head of the household is the husband, Rohingya women obey their husbands’ commands. We got such a case of a woman who had an implant for 5 years and after 2 months she came to us and cried to remove it as her husband forbade her to use it and wanted more children. The husband’s will is their will. Some women take pills temporarily and then husbands forbid they omit them.

(Supervisor of CHWs, Camp 14)

Almost all of the respondents lacked decision-making power regarding contraceptive use. Decisions regarding childbirth were primarily made by their husbands and family members, particularly their mother-in-law. Although a few respondents mentioned having the ability to express their opinions, follow-up questions revealed that their opinions were not valued at all. The majority of respondents confirmed that failing to comply with their husband's commands could result in the termination of their marriage.

I gave birth to my third child few months ago. A CHW sister came to my house and told me about the birth control. I told my husband. After listening to this, my husband was so angry that I won't say about it again. I don't want my marriage to end.

(A 27-year old woman, Camp 14)

The use of contraception in the camp can occasionally lead to gender-based violence. Women who secretly use contraception may be discovered and subsequently become victims of Intimate Partner Violence (IPV). The fear of facing violence and the potential for marital dissolution act as deterrents for women in utilizing contraceptives.

In the case of installing the device in IUD method, a thread comes out with the vaginal canal. It so happened that we installed the device in the afternoon and then the woman came back crying in the evening and said her husband beat her when watching this thread and sent her to remove it. If she doesn’t remove it, the marriage will end.

(A female medical officer, Camp 7)

### Role of religious beliefs

In the humanitarian context of Cox's Bazar, religion is recognized as a significant obstacle that hinders Muslim women from utilizing contraceptives. According to the respondents, not having the desired children is deemed as a sinful act and goes against the teachings of Islam. The use of birth control to reject what is perceived as a gift from Allah [God] is considered a sin and would displease Allah. Furthermore, these women perceive themselves as vessels responsible for the growth of the Islamic population in the world.

Children are gifts sent by Allah to fulfill some purpose. More children bring prosperity and happiness to the family. Killing the child means killing the gift that has been sent by the Allah and making him dissatisfied. (A mother of 4 daughters, Camp 14)

Children are given by Allah. We do not have these in our hands. We must accept obligingly what Allah will give. More children mean more Ummah of the prophet (sm). They will spread Islam all over the world. Allah's mercy is revealed in the family where there are more children.

(A 32-year old Rohingya woman, Camp 14)

Islamic principles, to a certain extent, discourage the utilization of contraception, particularly in cases where the mother is in good health and the family is capable of providing for additional children. However, the Rohingya community holds a more conservative perspective that deviates from the actual teachings of Islam.

They are very religious and abide by all Islamic rules. They think that it is forbidden to use birth control but the main thing is that if the mother’s health is in danger and the family has no ability to rear up any more child, it’s valid or acceptable to use birth control. They don’t know this. In their eyes, it is haram [*forbidden*] and very heinous work to do.

(A CHW, Camp 7)

### Fear of side effects

Respondents express reluctance to use contraceptive methods due to their fear of potential side effects associated with contraception. This fear is largely fueled by misconceptions and inaccurate information spread by friends and relatives. Commonly mentioned side effects include heavy bleeding, irregular menstruation, and body aches.

I heard from one of my female neighbors who used to take pills that she used to feel weak and dizzy after taking birth-spacing pills. She also experiences irregular menstrual cycles, body aches, heavy prolonged bleeding, etc. She told me she should not take the pills.

(A 24-year old woman from Camp7)

Findings from KIIs indicate that the fears expressed by Rohingya women regarding contraceptive use were not entirely baseless but had medical reasoning behind them. Female health specialists, in particular, reported a high occurrence of Dysfunctional Uterine Bleeding (DUB) among women who were unable to adhere to the prescribed pill regimen. According to participants, discontinuation, insufficient knowledge about proper usage of oral contraceptive pills (OCPs), and forgetfulness were commonly reported among Rohingya women. Since pills involve hormonal therapy, irregularities in their usage can lead to the occurrence of DUB. Women who experience DUB are more likely to share their experiences with others without fully comprehending the underlying cause, often attributing it to the contraceptives themselves.

They don’t take the pills regularly and can’t follow up. A sudden stop of hormonal therapy may cause imbalances in the body and irregularities in the menstrual cycle. Rohingya women come to us and cry about their bleeding. When we try to learn their history, we find that she took pills for 3 months but didn’t follow the directions. When such things happen, women think that it is because of contraceptives. They become afraid and demotivate other women to use it.

(A female medical officer, Camp 14)

### Misperceptions about contraception

The Rohingya community harbors deep-rooted stigma and misconceptions surrounding contraceptive methods. One of the most prevalent misperceptions is the belief that using contraceptives leads to infertility. This misconception serves as a primary deterrent, preventing many individuals from utilizing contraception.

My health collapsed after giving birth to my last son. I don’t want children now. CHW came to my home and told me about the birth control process. I have decided to take injections for 3 years but after talking to my husband about it, he forbids me. He said these injections are not good for health. They will make me unable to conceive in the future.

(A mother of 3 children, Camp 7)

KIIs corroborate the findings from Rohingya women, indicating that reluctance to use contraception stems from concerns about permanent sterility and potential health complications. Rumors spread within the camps when NGOs initially introduced contraceptives, further exacerbating the issue. Another prevalent misconception suggested that if a woman died with any contraceptive device inside her body, her funeral would not be accepted. This fear significantly discouraged the use of contraceptives among the Rohingya population.

When injectable contraceptives started, the rumor spread throughout the camp that if any woman take this contraceptive and wants to be pregnant in the future, the baby will be aborted automatically. Then to prevent this thing from happening another injection or totka [folk remedies] was invented by some quack or refugee doctor and spread throughout the camps. An interesting thing is that because of this antidote, the rate of abortion increased throughout the camp. Although we tried to track it down, we didn’t get any evidence.

(Information Management Officer, Camp 7)

Although some of the women take pills and depo, in terms of using long-term birth control, there is reluctance among them. Especially they don’t want to accept any device (Implant) because they think they if they die with this device, their funeral will never be accepted.

(A midwife, Camp-14)

Such rumors circulating within the Rohingya refugee community create fear and foster mistrust in the medical system, deterring individuals from utilizing vital family planning services.

## Discussion

This study employed a qualitative methodology to investigate the socio-cultural factors impacting the utilization of family planning methods among married Rohingya women in the humanitarian setting of Cox's Bazar, Bangladesh. Through the utilization of in-depth interviews (IDIs) and key informant interviews (KIIs), study participants shared insightful accounts of their experiences, perspectives on contraceptive usage, and factors contributing to non-usage. Socio-cultural values such as the desire for more children to maintain the family line and to be able to contribute to the growth of the Islamic population pose a significant obstacle. Women's limited control over reproductive choices not only hinders their involvement but also exposes them to IPV and marriage dissolution within the camps. Religious beliefs view contraceptives as sinful and valuing children as divine blessings further promote larger families. Moreover, the fear of side effects stems from myths, misconceptions, and mistrust in the existing medical system also identified as significant challenges. These findings provide a valuable comprehension of the sociocultural influences shaping the utilization of family planning services within this vulnerable population. Policymakers can harness this knowledge to design culturally sensitive interventions that may find relevance in similar contexts as well.

The study finding reported that Rohingya women are inclined to use family planning methods because of their willingness to have more children. This finding corroborates with other study findings conducted in the same setting [15]. The reasons for wanting more children can be attributed to various factors, including a preference for male offspring [19], the expectation of financial protection and care in old age [25, 26], the belief that children are blessings from God associated with prosperity [19, 27] the aim to contribute to the growth of the Islamic population, the expectation of better chances of survival in refugee camps [28, 29] and the hope of getting additional food cards with benefits such as food, medicine, and clothes [15]. In addition, another significant reason for desiring children is rooted in traditional gender roles, which expect women to become mothers shortly after marriage and consider motherhood as the primary responsibility in a woman’s life [17, 30].

The present study also found that although a small number of respondents expressed willingness to use contraception, the main reason for their non-use was the disapproval of their husbands. This finding is consistent with other studies conducted both in refugee and non-refugee settings, highlighting husband disapproval as a key factor preventing women from using contraception [31–33]. The decision of accessing reproductive health services, including contraception, is typically made by husbands and mothers-in-law [15, 17, 19]. The main reasons for the disapproval were identified as the husband’s desire for more children [34, 35], religious belief [36], the social stigma associated with contraceptive use [31, 37], and misperception regarding the side effects of contraceptives [31, 34]. Religiously and culturally, Rohingya women believe that engaging in any activity without their husband’s permission is considered a sin [15]. This belief justifies husbands' actions, regardless of their appropriateness.

Consistent with another study, the finding of this study revealed that Rohingya women often obey their husbands’ commands in matters of contraception, fearing repercussions such as intimate partner violence or the threat of ending marital relations [38]. Previous studies validate the existence of a prominent patriarchal social structure among the Rohingya community [17, 19, 27]. The prevalent social and religious norms of this community hold women to be responsible for the household, children, and domestic care work; constrain women’s mobility, and foster dependency on men [17, 19]. Consequently, men gain additional power to make decisions, leading to power imbalances and curtailed decision-making capacity for women. Similarly, the cultural expectation of submissive and patient women, juxtaposed with strong and aggressive men, validates and reinforces the improper exercise of power, for instance, IPV, by men [39]. Moreover, the normalization of violence by men within the culture saying that “man has a right to punish a woman” Or “if a man beats his wife, it shows that he loves he” [40] validates and encourages future perpetration [41]. These norms and power imbalances between spouses made women hesitate to talk to their husbands about family planning and limited their decision-making power regarding contraception [42, 43]. Our findings indicate that the involvement of men in family planning can enhance both the uptake and continuation of contraceptive use. Therefore, to enhance contraception rates, interventions should educate and raise awareness among both women and men about family planning and encourage open communication between partners about contraceptive methods.

Strong religious belief among Rohingya women serves as a barrier to contraceptive use and access to other SRH services [44]. Research has confirmed that Rohingyas are culturally and religiously conservative, relying on religion as a means to interpret various aspects of their lives [19, 26, 45]. The practice of Purdah, restricted mobility, and restrictions on interacting with men outside of the immediate family significantly limit their chances to access many of the available services [19, 45]. Moreover, their belief that contraception is against the tenet of Islam and using it is a sin which will dissatisfy Allah (God) directly prevents them from using contraception. This belief is also widespread among the Muslim population worldwide [36]. In contrast, studies on Syrian women in the refugee camp in Jordan and Lebanon indicated that religion was not considered a barrier for married Syrian women in accessing family planning services [46, 47]. Furthermore, many religious leaders believe that Islam encourages the practice of family planning [48]. Muslim nations, including Bangladesh, are actively advocating for maternal and child health, including modern contraception, which encompasses the promotion of modern contraception methods [37, 49]. Therefore, to increase the acceptance of contraceptives, it is recommended to promote accurate information through awareness campaigns. Additionally, religious leaders from the Rohingya community should be engaged in advocacy work to disseminate correct information.

Fear of side effects was mentioned by a significant number of the Rohingya women and the stakeholders as a major reason for the non-use of contraception, as has been confirmed in both refugee and non-refugee settings [31, 32, 34]. The most commonly reported side effects were weight gain, headaches, body aches, excessive and prolonged bleeding, and irregular menstruation [31, 50]. Although the scientific evidence points to lesser side effects [34], these can be managed medically [51] and the benefits such as powerful protection against several life-threatening diseases and increased life expectancy outweigh the risks [52, 53]. Moreover, studies have reported that many of these side effects can be caused by the low quality of care, incorrect positioning or displacement of contraceptive devices, lack of follow-up treatments, lack of proper pre and post-counseling, and not following the proper rules mentioned by the healthcare providers [50, 54, 55]. For instance, a study on Implant removal experiences among Ethiopian women found that participants experienced arm aches and discomfort in the insertion area because of incorrect positioning [54]. The current study found that Rohingya women face difficulties in remembering the rules of taking oral contraceptive pills which causes inconsistency in routine and impacts their menstrual cycle [56]. Thereby, the finding indicates the importance of counseling programs both before and following contraceptive insertion, to inform women of the benefits and the potential side effects. It is essential to appoint qualified healthcare providers with adequate knowledge, hands-on training, and experience for the insertion process. Additionally, it is recommended to initially promote more acceptable alternatives for women, such as the rhythm and withdrawal methods, which have been proven to be simpler, safer, and congruent with cultural beliefs [34].

Similar to our findings, studies conducted in refugee camps of Nigeria [57], Thailand [32], Jordan [47], and Lebanon [46] confirmed that misperceptions about family planning methods play a much greater role in decision-making. The most cited misperception was the use of such methods can cause permanent infecundity and birth defects and abnormalities [31, 57]. Another prevailing misperception among the Rohingya population is that healthcare providers intentionally provide contraception to induce sterilization, thus preventing them from permanently settling in Bangladesh [58]. In a study conducted by Zerihun et al. [54] found that religious leaders perceive the funeral of a woman who dies with an implant as incomplete and delay the burial for removing it, which is one of the reasons Ethiopian Muslim women opt for early removal of the implant [54]. These misperceptions persist among refugees prior to their displacement [32] and are perpetuated across generation through a few channels [35, 59]. According to our study, Rohingya women primarily rely on their female friends, family members, and neighbors as sources of information related to family planning. Evidence indicates that peers and other social networks are highly effective in disseminating rumors, misinformation, and exaggerated accounts of rare side effects [31, 35, 59]. A study on the Oru refugee camp also reported low use of contraceptives among young refugees because of the different misconceptions, negative perceptions, and rumors [57]. The findings stress the need to create mass and peer campaigns to engage a wider community in various family planning programs. This campaign should specifically target women, addressing prevalent myths and misconceptions while promoting awareness about family planning.

The study has various limitations. Firstly, it did not incorporate the viewpoints of Rohingya men, which could have enriched the comprehensive understanding of the findings. Secondly, the data collection was confined to just two unregistered camps due to mobility restrictions imposed by the COVID-19 pandemic. Consequently, the outcomes might not completely represent the perspectives of the intended population. Moreover, there exists the potential for certain nuances to have been lost during the translation process from Rohingya to Bangla and then to English. To mitigate this concern, we took steps to minimize errors by involving experienced data collectors and two distinct groups of transcribers fluent in the Chittagonian dialect of Bengali, which closely mirrors the Rohingya language.

## Conclusion

The study revealed that socio-cultural and religious beliefs were constraining the use of contraceptives among Rohingya refugees. Rohingya women who actively sought pregnancies displayed reluctance towards contraceptive methods. Societal norms, such as early marriage post-puberty, immediate motherhood after marriage, and the desire for a larger number of children to strengthen lineage, acted as barriers to contraception. Moreover, the limited agency of women in decisions concerning their reproductive lives led to an imbalance in power dynamics, contributing to an increase in IPV within the camps. Religious convictions that view contraceptives as sinful and consider children as a divine gift further encouraged the community to pursue larger families. Hindrances such as concerns about side effects and the propagation of misconceptions through the Rohingya social network compounded the issue.

Based on our findings, there is a pressing need to establish mass and peer-driven campaigns that involve the wider community in diverse family planning initiatives. These campaigns should particularly focus on women and their husbands, addressing prevalent myths and misconceptions while simultaneously enhancing awareness regarding family planning. Furthermore, counseling programs facilitated by skilled healthcare providers should be introduced before and after contraceptive insertion, aiming to educate women about the benefits and potential side effects. Additionally, involving religious leaders from the Rohingya community in advocacy efforts is crucial for the accurate dissemination of information.

## Data Availability

All of the primary data has been included in the results. Additional materials with details may be obtained from the corresponding author if required.
